# A Systems Pharmacology Model of Aging Identifies Optimal Combination Therapies With Secondary Benefits on Weight Loss and Metabolic Health

**DOI:** 10.1002/psp4.70322

**Published:** 2026-08-10

**Authors:** Igor Goryanin, Bob Damms, Irina Goryanin

**Affiliations:** ^1^ IQANOVA Ltd Edinburgh UK; ^2^ School of Informatics University of Edinburgh Edinburgh UK

**Keywords:** aging, Bayesian meta‐analysis, combination therapy, GLP‐1 receptor agonists, identifiability, model‐informed drug development, quantitative systems pharmacology, rapamycin, SBML

## Abstract

Aging is a systems‐level process linking metabolic dysfunction, inflammation, impaired repair, frailty, and multimorbidity, whereas existing pharmacological strategies usually optimize disease‐specific endpoints such as weight loss or HbA1c rather than aging‐related trajectories. We developed an SBML‐compliant quantitative systems pharmacology (QSP) model in which aging is represented as a dynamic, pharmacologically modifiable endpoint. The model integrates four coupled layers: metabolic/pharmacodynamic responses to GLP‐1 receptor agonism, SGLT2 inhibition, metformin and rapamycin; adverse‐event dynamics; aging states including damage accumulation, repair capacity, frailty and biological age gap; and biomarker outputs including GDF15, cystatin C, leptin, adiponectin and estimated glucose disposal rate. The semaglutide submodel was calibrated against published STEP trial endpoints, and Bayesian hierarchical meta‐analysis, global sensitivity analysis, practical identifiability analysis and internal consistency checks were used to assess model behavior. The calibrated model reproduced semaglutide‐associated weight loss, HbA1c reduction and transient nausea within pre‐specified error benchmarks. Bayesian meta‐analysis confirmed strong metabolic effects for semaglutide, moderate glycaemic effects for SGLT2 inhibitors and metformin, and a near‐zero HbA1c effect for rapamycin. Sensitivity analysis revealed largely orthogonal metabolic and aging parameter spaces. Combination simulations identified two mechanistically distinct optima: GLP‐1 receptor agonist plus SGLT2 inhibitor plus metformin for metabolic improvement, and GLP‐1 receptor agonist plus SGLT2 inhibitor plus rapamycin for aging‐related benefit. Metabolic optimisation and aging optimisation are therefore mechanistically distinct objectives that do not converge on the same drug combination. These predictions are hypothesis‐generating and require external validation against independent longitudinal datasets and clinical safety evaluation before translation to treatment recommendations.

## Study Highlights

- What is the current knowledge on the topic?
  ○ Aging contributes to multimorbidity through interacting metabolic, inflammatory, nutrient‐sensing, repair and frailty pathways. GLP‐1 receptor agonists, SGLT2 inhibitors, metformin and rapamycin affect parts of this biology, but are usually assessed using disease‐specific endpoints.
- What question did this study address?
  ○ Can an SBML‐compliant QSP model represent aging as a pharmacologically modifiable endpoint and distinguish combinations optimized for metabolic benefit from those optimized for aging‐related benefit?
- What does this study add to our knowledge?
  ○ The model integrates metabolic pharmacodynamics, adverse‐event dynamics, damage accumulation, repair capacity, frailty, biological age gap and biomarkers. It predicts that GLP‐1 RA + SGLT2 inhibitor + metformin is the metabolic optimum, whereas GLP‐1 RA + SGLT2 inhibitor + rapamycin is the aging‐oriented mechanistic optimum.
- How might this change drug discovery, development and/or therapeutics?
  ○ The framework supports model‐informed prioritization of healthspan‐directed combinations, biomarker panels beyond weight and HbA1c, and testable trial designs for aging‐related therapeutic hypotheses, pending external validation and safety evaluation.

## 1 Introduction

Aging is the dominant common risk factor for most chronic diseases and functional decline. Type 2 diabetes, cardiovascular disease, chronic kidney disease, sarcopenia and frailty frequently co‐occur because they emerge from overlapping biological disturbances rather than isolated organ‐specific failures. At the systems level, aging is associated with cumulative molecular and cellular damage, impaired nutrient sensing, metabolic inflexibility, chronic low‐grade inflammation and declining repair capacity [1].

Metabolic dysfunction is a particularly important amplifier of aging biology. Chronic hyperglycaemia, insulin resistance and excess adiposity accelerate tissue damage and promote cumulative biological burden. Biomarkers including GDF15, cystatin C, leptin, adiponectin, HbA1c and estimated glucose disposal rate (eGDR) can therefore be interpreted not only as disease‐associated measurements but also as system‐level readouts of biological aging, metabolic burden and physiological reserve [2, 3, 4, 5].

Current pharmacological strategies remain organized around conventional disease boundaries. GLP‐1 receptor agonists achieve substantial weight loss and glycaemic control through incretin‐mediated pathways [6, 7, 8]. SGLT2 inhibitors improve cardiometabolic, cardiovascular and renal outcomes [9, 10, 11, 12, 13]. Metformin modulates metabolic pathways via AMP‐activated protein kinase (AMPK) activation [14]. Rapamycin and rapalogues target mechanistic target of rapamycin (mTOR), a central nutrient‐sensing pathway linked to aging biology [15, 16]. These agents are rarely integrated within a unified mechanistic framework that explicitly asks how their combined actions reshape damage accumulation, repair, frailty and biological age trajectories.

Quantitative systems pharmacology (QSP) provides a natural platform for this problem because it links drug action, physiology, adverse events and downstream biomarkers within a causal structure. Existing incretin and metabolic QSP models have provided important foundations for modeling glucose control, incretin pharmacology and body‐weight dynamics [17, 18, 19], but they typically stop short of representing aging as a formal state variable or primary therapeutic endpoint.

Here we present an aging‐centered, SBML‐compliant mechanistic QSP model calibrated against published clinical trial data and subjected to Bayesian hierarchical meta‐analysis, global sensitivity analysis and practical identifiability analysis. We hypothesized that (i) aging can be represented as a dynamic pharmacologically modifiable systems process; (ii) biomarker‐informed calibration can constrain biological‐age trajectories; and (iii) rationally selected combination therapies may produce mechanistically distinct metabolic and healthspan‐oriented optima.

## 2 Methods

### 2.1 Model Structure and Context of Use

**FIGURE 1:**
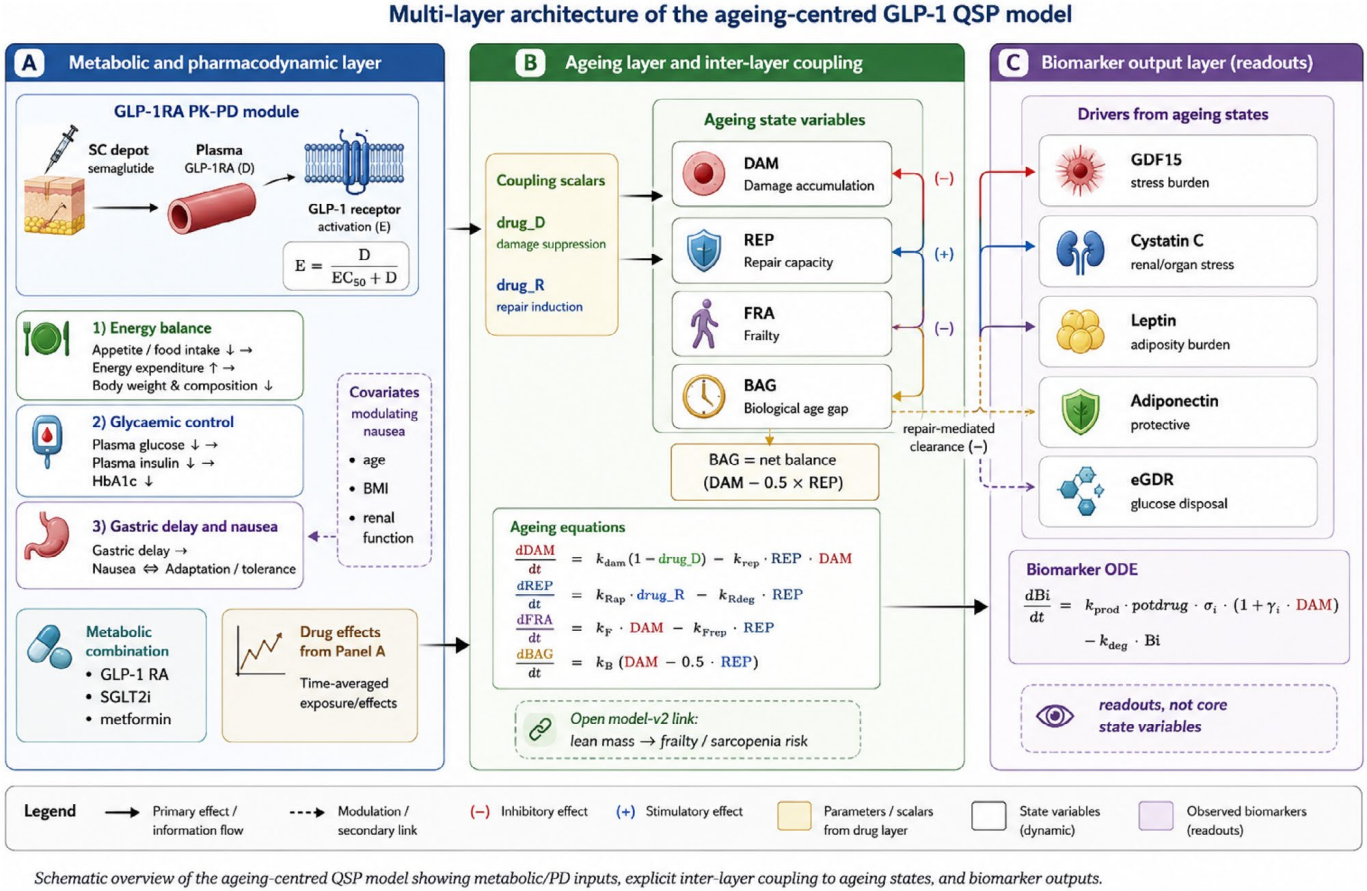
Structure of the GLP‐1/semaglutide quantitative systems pharmacology model. The semaglutide pharmacokinetic compartment describes subcutaneous absorption and plasma clearance to yield plasma GLP‐1 receptor agonist concentration. GLP‐1 receptor activation distributes drug effect to the energy‐balance module, which includes appetite, food intake, energy expenditure, fat mass and lean mass, and to the glycaemic‐control module, which includes plasma glucose, insulin and HbA1c. The gastric‐delay and nausea module encodes tolerance‐mediated transient nausea, with patient covariates including age, body mass index and renal function.

The model was designed for hypothesis generation and mechanistic prioritization of candidate combination therapies for healthspan‐related endpoints in adults with obesity and/or type 2 diabetes. It is not intended to support definitive Phase II/III treatment selection or regulatory decision‐making without external validation against independent longitudinal datasets. The model consists of four interacting mechanistic layers: a metabolic/pharmacodynamic layer, a drug‐effect layer, an adverse‐event layer and an aging/biomarker layer. Figure 1 shows the semaglutide metabolic, glycaemic and nausea architecture; the aging‐state and biomarker equations are specified in the following sections.

### 2.2 Scientific Rationale for Model Structure

The logistic approach‐to‐maximum ODEs for weight and HbA1c were chosen to encode clinically observed saturation of semaglutide effect and the physiological constraint that weight loss cannot continue indefinitely. The nausea module includes an adaptation/tolerance state because STEP trials show a peak‐and‐decline pattern of gastrointestinal adverse events despite continued exposure. The aging layer uses a damage–repair formalism consistent with the frailty‐as‐deficit framework and the hallmarks‐of‐aging concept, where damage accumulation and declining resilience jointly determine functional vulnerability [1, 20, 21]. Biological age gap is represented as a net balance between damage and repair rather than as a direct surrogate of weight or HbA1c.

### 2.3 Layer Coupling Architecture and Inter‐Layer Equations

Layer 1 produces effective drug exposure E, computed using a Hill‐type saturation function of administered dose D. Drug exposure enters the aging layer through two scenario‐defined scalars: drug_D, representing damage suppression, and drug_R, representing repair induction. Layer 2 aging states then drive Layer 3 biomarker production through damage‐sensitive and repair‐sensitive output terms.

$$E=D/\left(\mathrm{EC}50+D\right)$$

$$\text{dDAM}/\mathrm{dt}=k\_\mathrm{dam}\cdot \left(1-\text{drug}\_D\right)-k\_\mathrm{rep}\cdot \mathrm{REP}\cdot \mathrm{DAM}$$

dREP/dt=k_Rap·drug_R−k_Rdeg·REP

dFRA/dt=k_F·DAM−k_Frep·REP

$$\text{dBAG}/\mathrm{dt}=k\_B\cdot \left(\mathrm{DAM}-0.5\cdot \mathrm{REP}\right)$$

$$\mathrm{dBᵢ}/\mathrm{dt}=k\_\text{prodᵢ}\cdot \mathrm{pot}\_\text{drug}\cdot \mathrm{\sigma ᵢ}\cdot \left(1+\mathrm{\gamma ᵢ}\cdot \mathrm{DAM}\right)-k\_\text{degᵢ}\cdot \mathrm{Bᵢ}$$

For the metabolic combination (GLP‐1 RA + SGLT2 inhibitor + metformin), drug_D = 0.40 and drug_R = 0.10. For the aging‐oriented combination (GLP‐1 RA + SGLT2 inhibitor + rapamycin), drug_D = 0.50 and drug_R = 0.60. These values are scenario inputs used to compare mechanistic hypotheses, not claimed clinical effect sizes.

### 2.4 Metabolic and Pharmacodynamic Layer

Semaglutide‐driven body‐weight change (W, % from baseline) and HbA1c reduction (A, % absolute) were modeled using logistic approach‐to‐maximum ordinary differential equations. The same exposure function E was used for weight and HbA1c modules, with separate rate constants and maximum effects. Nausea prevalence (N, %) was modeled with an adaptation state T: dN/dt = k_N · E · (1 − T) − 0.15 · N and dT/dt = k_T · N · (1 − T), representing tolerance‐mediated resolution after early peak nausea [6, 7].

### 2.5 Drug‐Specific Pharmacodynamic Implementations

GLP‐1 receptor agonism was implemented through the exposure–response structure described above, informed by incretin and glucose‐regulation QSP literature [17, 18, 19] and STEP trial trajectories [6, 7, 8]. Metformin was modeled as an AMPK‐associated metabolic modulator that reduces damage accumulation and provides modest repair support [14]. SGLT2 inhibition was modeled as reducing metabolic‐glycaemic stress and glucotoxicity, consistent with large cardiovascular and renal outcome trials [9, 10, 11, 12, 13]. Rapamycin/rapalogues were modeled as direct repair‐capacity inducers through mTOR inhibition [15, 16]. Sodium‐fluid homeostasis, renal haemodynamic modulation and heart‐failure‐specific SGLT2 mechanisms are outside the current model scope and would be required before application to CKD or heart‐failure populations.

### 2.6 Aging and Biomarker Output Layers

The aging layer incorporated four coupled state variables: damage accumulation (DAM), repair capacity (REP), frailty (FRA) and biological age gap (BAG). Frailty was driven by damage and suppressed by repair capacity, consistent with frailty as a resource‐deficit and vulnerability state [20, 21]. Five biomarker outputs were modeled as first‐order production–degradation readouts: GDF15, cystatin C, leptin, adiponectin and eGDR. GDF15 and cystatin C serve as stress and renal‐metabolic burden markers; leptin tracks adiposity‐related burden; adiponectin and eGDR reflect favorable metabolic function and repair‐associated resilience [2, 3, 4, 5].

### 2.7 SBML Implementation

The complete model was encoded in SBML Level 3 Version 2 (model ID: IQANOVA_GLP1_Aging_QSP) with MIRIAM‐compatible metadata, SBO‐oriented reaction terms, structured kinetic laws and complete unit definitions. Structural validation using libSBML confirmed zero fatal errors, zero errors and zero warnings across the model species, parameters, reactions and assignment rules [22].

### 2.8 Synthetic Calibration Dataset Generation

Synthetic calibration data were constructed by digitizing published STEP 1 and STEP 2 trial figures and adverse‐event tables at seven time points (0, 4, 12, 24, 36, 52 and 68 weeks) [6, 7]. For body weight and HbA1c, mean values and 95% confidence intervals were extracted from primary trial figures; standard errors were propagated as SE = (upper CI − lower CI)/(2 × 1.96). For nausea prevalence, event proportions and trial‐arm denominators were extracted from adverse‐event tables, with SE computed as √(*p*(1 − *p*)/*N*). Interpolation used monotone cubic splines constrained to reproduce the observed peak‐and‐decline pattern of nausea. The complete dataset is provided as all_synthetic_data.csv.

### 2.9 Staged Calibration Strategy and Provenance

Calibration followed a three‐stage workflow. Stage 1 calibrated semaglutide pharmacodynamic and adverse‐event layers against STEP 1 and STEP 2 trajectories [6, 7]. Stage 2 anchored aging‐state and biomarker layers using literature‐derived population targets: frailty trajectory shape from Clegg and Fried frameworks [20, 21], rapamycin/rapalogue repair induction from Mannick and PEARL data [15, 16], metformin damage modulation from metabolic intervention literature [14, 23, 24], and biomarker targets from multimorbidity, GDF15, kidney and eGDR studies [2, 3, 4, 5]. Stage 3 performed integrated recalibration with the highest weights assigned to clinical semaglutide endpoints and lower weights assigned to surrogate aging outputs. This is explicitly a surrogate‐integrated calibration, not an individual‐level longitudinal ODE refit.

### 2.10 Pre‐Specified RMSE Benchmarks

RMSE benchmarks were set before model fitting and differed by endpoint because the endpoints have different measurement scales and uncertainty structures: body weight < 1.0% change from baseline, HbA1c < 0.15% absolute, and nausea prevalence < 5% absolute. These thresholds reflect clinically meaningful body‐weight differences, HbA1c assay precision and binomial uncertainty in self‐reported adverse‐event prevalence rather than arbitrary uniform error limits [19].

### 2.11 Bayesian Hierarchical Meta‐Analysis

A Bayesian random‐effects hierarchical meta‐analysis was conducted across 37 published trial‐arm observations to derive drug‐class pooled effect estimates for body‐weight change, HbA1c reduction and nausea prevalence. Studies included STEP 1–5, SUSTAIN 6, SELECT, PIONEER 6 and SURPASS‐2 for incretin‐related comparators; EMPA‐REG OUTCOME, DECLARE–TIMI 58, DAPA‐HF, CREDENCE and CANVAS for SGLT2 inhibitors; UKPDS and DPP for metformin; and Mannick/PEARL rapamycin or rapalogue studies [6, 7, 8, 9, 10, 11, 12, 13, 14, 15, 16, 23, 24, 25, 26, 27, 28, 29, 30]. Priors followed weakly informative recommendations for hierarchical variance parameters [31]. Sampling used the No‐U‐Turn Sampler implemented in PyMC, with convergence assessed using *R̂* and effective sample size [32]. Between‐study heterogeneity was quantified using I^2^ [33].

### 2.12 Global Sensitivity and Identifiability Analyses

Global sensitivity analysis used Latin Hypercube Sampling (*N* = 3000) over ±30% variation around nominal values, a physiologically plausible exploratory QSP range [34]. The main analysis varied 11 primary pharmacodynamic and aging‐layer parameters. Fixed literature constants, output‐only biomarker parameters and scenario‐defined drug scalars were documented separately; a supplementary extended GSA varied biomarker‐layer parameters and confirmed that primary endpoint rankings were unchanged. For weakly identified nausea parameters, a ±50% robustness analysis was performed.

Practical identifiability was assessed using the Fisher Information Matrix and profile likelihood. Relative standard errors were derived from the Cramér–Rao lower bound and classified as well identified (< 20%), moderately identified (20%–50%) or weakly identified (> 50%). Profile‐likelihood confidence intervals were computed using established likelihood‐threshold methods [35, 36, 37].

### 2.13 Quantitative Limitation‐Bounding Analysis

To quantify two omitted biological domains without implying additional calibration or validation, we performed a deterministic bounding analysis. The fraction of total weight loss attributable to lean mass (f_LM) was varied from 0 to 0.40, spanning the range reported across semaglutide clinical studies [38], and implied lean‐mass change was calculated as f_LM × |ΔW|. For epigenetic translation, the normalized model BAG was mapped through BAG_epi = s_epi × BAG + *ε*, with a transfer‐slope bound s_epi = 0.5–1.5 and a minimum post‐mapping uncertainty band of ±3.6 years, corresponding to the median error reported for the multi‐tissue Horvath clock [39]. These quantities are uncertainty bounds, not fitted model parameters or validation results.

## 3 Results

### 3.1 Clinical Model Performance and Calibration

**FIGURE 2:**
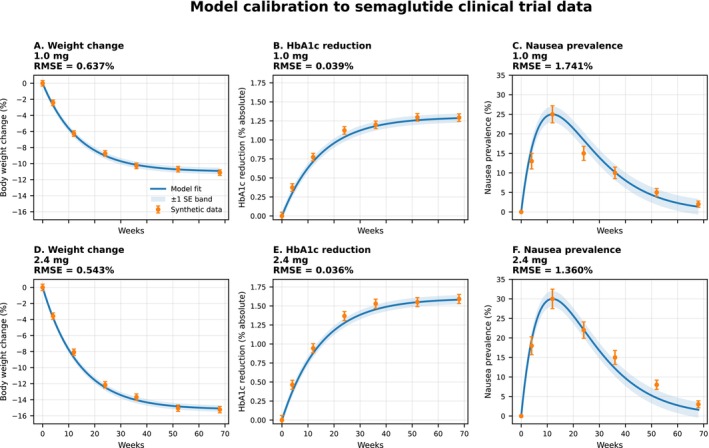
Model calibration to semaglutide clinical trial data. Synthetic calibration data (filled circles ±1 SE) anchored to STEP 1 and STEP 2 are shown with model‐fit curves and ±1 SE bands. The six panels present body‐weight change, HbA1c reduction and nausea prevalence for the 1.0 mg and 2.4 mg arms. All RMSE values were within the pre‐specified benchmarks: Body weight < 1.0%, HbA1c < 0.15% absolute and nausea prevalence < 5% absolute.

The calibrated model reproduced the expected qualitative and quantitative pattern of semaglutide response across all three primary clinical endpoints (Figure 2). Dose‐dependent weight loss was captured with RMSE of 0.637% in the 1.0 mg arm and 0.543% in the 2.4 mg arm. HbA1c reduction was reproduced with RMSE of 0.039% and 0.036%, and nausea prevalence was reproduced with RMSE of 1.741% and 1.360%. All values were within pre‐specified benchmarks.

### 3.2 Bayesian Meta‐Analysis: Drug‐Class Effect Estimates

**FIGURE 3:**
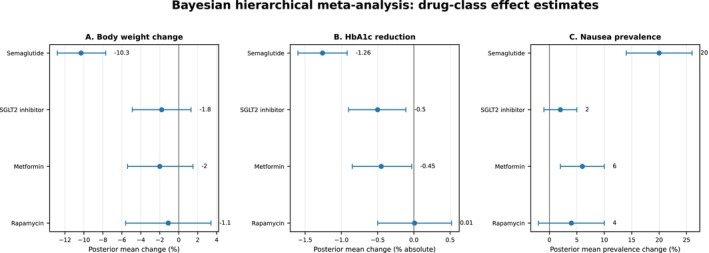
Bayesian hierarchical random‐effects meta‐analysis across 37 trial‐arm observations. Forest plots show individual study estimates and pooled drug‐class estimates with 95% credible intervals for body‐weight change, HbA1c change and nausea prevalence. The analysis supports strong metabolic effects of semaglutide, moderate glycaemic effects of SGLT2 inhibitors and metformin, and a near‐zero HbA1c effect for rapamycin.

The Bayesian hierarchical meta‐analysis yielded well‐converged posterior distributions for all drug classes (*R̂* ≤ 1.001; minimum effective sample size > 1900). Semaglutide produced the largest posterior body‐weight reduction (−10.3%, 95% CrI −12.8 to −7.7), exceeding the SGLT2 inhibitor, metformin and rapamycin/rapalogue classes. For HbA1c, semaglutide, SGLT2 inhibitors and metformin showed credible glycaemic improvements, whereas rapamycin had a near‐zero posterior effect (+0.006%, 95% CrI −0.50 to +0.52), supporting separation between glycaemic and repair‐oriented mechanisms (Figure 3; Tables 1 and 2).

**TABLE 1:** Bayesian hierarchical meta‐analysis—posterior drug‐class effect estimates.

Drug class	Endpoint	*k*	Posterior mean	95% CrI	*p* (direction)
Semaglutide	Weight (%)	6	−10.31	[−12.82, −7.70]	1.000
SGLT2 inhibitor	Weight (%)	4	−1.84	[−4.92, +1.27]	0.890
Metformin	Weight (%)	3	−1.97	[−5.42, +1.53]	0.882
Rapamycin/rapalogues	Weight (%)	2	−1.15	[−5.59, +3.36]	0.706
Semaglutide	HbA1c (%)	4	−1.26	[−1.60, −0.92]	1.000
SGLT2 inhibitor	HbA1c (%)	3	−0.50	[−0.90, −0.11]	0.989
Metformin	HbA1c (%)	3	−0.45	[−0.85, −0.03]	0.981
Rapamycin/rapalogues	HbA1c (%)	2	+0.006	[−0.50, +0.52]	0.488
Semaglutide	Nausea (%)	4	38.6	[33.7, 43.0]	High
Metformin	Nausea (%)	2	19.7	[13.7, 25.6]	—
Rapamycin/rapalogues	Nausea (%)	2	7.8	[2.2, 13.9]	—
SGLT2 inhibitor	Nausea (%)	2	5.6	[0.3, 11.3]	—

*Note:* NUTS sampler; 4 chains × 1500 draws; *R̂* ≤ 1.001; minimum effective sample size > 1900. Heterogeneity was quantified using *I* ^2^ and *τ*.

**TABLE 2:** Parameter identifiability summary (Cramér–Rao relative standard error).

Parameter	Layer/model	Nominal value	Units	RSE (%)	Class	Interpretation/recommendation
Emax_A	Semaglutide PD	1.80	%	11.1	Well	No action needed
k_W	Semaglutide PD	0.20	wk^−1^	33.8	Moderate	Refine with STEP 3/4/5 trajectories
Emax_W	Semaglutide PD	15.0	%	48.8	Moderate	Plateau characterized; correlation with k_W remains
k_A	Semaglutide PD	0.15	wk^−1^	46.1	Moderate	Accept for hypothesis generation; refine with additional HbA1c data
k_N	Nausea module	0.35	wk^−1^	184.1	Weak	Fix at literature value; weekly nausea data required
k_T	Nausea module	0.04	wk^−1^	169.7	Weak	Collinear with k_N; reparameterise tolerance ratio
EC50	Exposure function	2.0	mg	82.1	Weak	Fixed from literature in scenario simulations
k_dam	Aging layer	0.10	wk^−1^	1.5	Well	Locally well constrained under surrogate‐integrated calibration
k_rep	Aging layer	0.80	wk^−1^	2.1	Well	Locally well constrained
k_Rdeg	Aging layer	0.25	wk^−1^	0.7	Well	Locally well constrained
k_Rap	Aging layer	0.45	wk^−1^	1.2	Well	Top GSA driver for REP and BAG

*Note:* Low RSE values for aging‐layer parameters should be interpreted as internal local identifiability under surrogate‐integrated calibration, not as proof of practical estimability from future individual‐level clinical datasets.

### 3.3 Global Sensitivity Analysis

**FIGURE 4:**
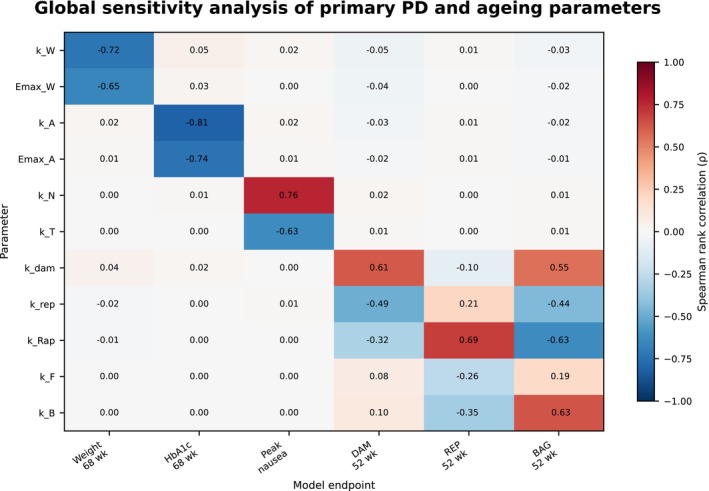
Global sensitivity analysis using 3000 Latin Hypercube Sampling realizations and ±30% parameter variation. Heatmap values are Spearman rank correlations between model parameters and endpoints. The analysis separates metabolic/tolerability drivers from aging drivers: K_rapamycin dominates repair capacity and biological age gap while contributing negligibly to body weight or HbA1c.

GSA revealed a clear separation between metabolic and aging parameter spaces (Figure 4). k_weight dominated body weight, k_HbA1c dominated HbA1c, and nausea was governed by competing induction and tolerance parameters. By contrast, k_Rap was the dominant driver of repair capacity and biological age gap while contributing negligibly to body weight or HbA1c, providing the mechanistic basis for the two‐optima finding.

### 3.4 Parameter Identifiability

All aging‐layer parameters were locally well identified under the surrogate‐integrated calibration structure, with RSE values below 3%. Semaglutide HbA1c plateau was also well identified; k_W and k_A were moderately identified; k_N, k_T and EC50 were weakly identified because of collinearity between nausea induction, adaptation and exposure. Weak nausea parameters were therefore fixed at literature‐derived values in combination simulations and tested over wider sensitivity ranges.

### 3.5 Biomarker Trajectories

The biomarker output layer generated internally coherent directional predictions across all five outputs (Figure S2). GDF15 and cystatin C tracked stress and renal‐metabolic burden; leptin reflected adiposity‐related burden; and adiponectin and eGDR improved under treatment, consistent with favorable metabolic and repair‐associated states. These trajectories provide translational readouts for prospective evaluation but are not independently validated clinical predictions.

### 3.6 Combination Therapy Simulations

**FIGURE 5:**
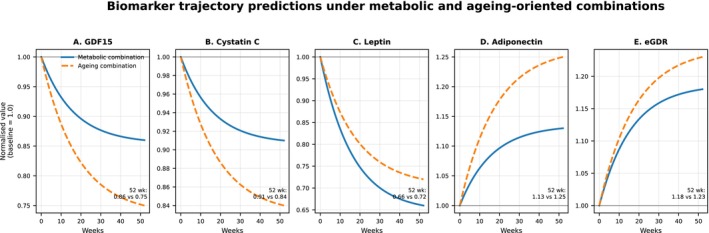
Combination‐therapy simulations predict distinct aging outcomes at 52 weeks. GLP‐1 receptor agonist + SGLT2 inhibitor + metformin is compared with GLP‐1 receptor agonist + SGLT2 inhibitor + rapamycin. The rapamycin‐containing combination produces larger reductions in damage accumulation, frailty and biological age gap and a larger increase in repair capacity; these are model‐based mechanistic hypotheses rather than clinical treatment recommendations.

**FIGURE 6:**
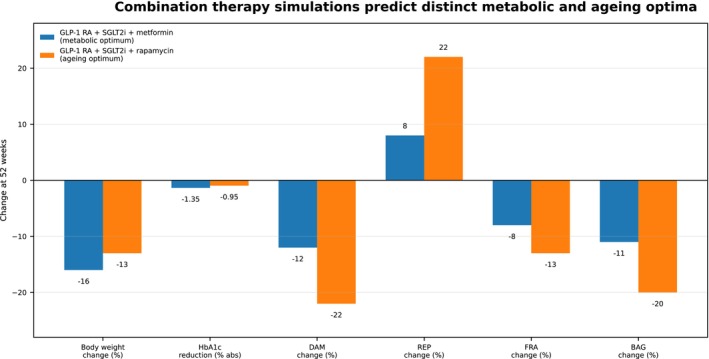
Combination therapy simulations predict distinct metabolic and aging optima.

Combination simulations identified two mechanistically distinct therapeutic optima (Figure 5). GLP‐1 RA + SGLT2 inhibitor + metformin produced the strongest metabolic profile, with strong weight loss and glycaemic improvement. GLP‐1 RA + SGLT2 inhibitor + rapamycin produced larger reductions in damage accumulation, frailty and biological age gap and a larger increase in repair capacity (Figure 6). The latter result reflects rapamycin‐driven repair induction rather than superior metabolic control and should be interpreted as a mechanistic hypothesis rather than a clinical recommendation.

## 4 Discussion

### 4.1 Aging as a Pharmacological Target

This study reframes metabolic pharmacotherapy within an aging‐centered systems framework. The principal finding is that optimal metabolic control and optimal aging modulation are mechanistically distinct objectives that do not necessarily converge on the same drug combination. This interpretation is consistent with the hallmarks‐of‐aging framework, where nutrient sensing, damage accumulation, repair capacity and resilience represent partially separable biological axes [1].

### 4.2 Clinical Implications and the Two‐Optima Framework

The two‐optima result has practical implications for trial design. If weight loss and HbA1c are used as the only decision criteria, the metabolic optimum will be favored. If the objective is healthspan‐related biology, biomarkers of damage, repair, frailty and metabolic reserve must also be measured. The model therefore supports prospective testing of biomarker panels including GDF15, cystatin C, adiponectin and eGDR alongside standard metabolic endpoints [2, 3, 4, 5].

### 4.3 Drug Interaction Topologies and Additivity Assumptions

The base‐case model uses scenario‐defined drug effect scalars and therefore approximates combination effects as additive. This is a simplifying assumption. AMPK–mTOR cross‐talk, incretin‐mediated changes in appetite and metabolism, and SGLT2i‐mediated changes in systemic stress may generate subadditive or synergistic interactions. Compares additive, subadditive pathway‐saturation and Bliss‐style interaction assumptions; the qualitative aging advantage of the rapamycin‐containing arm is preserved, although the magnitude of BAG improvement is attenuated under subadditivity.

### 4.4 Dosing Complexity and Intermittent Rapamycin

The present model uses time‐averaged drug_D and drug_R scalars over a 52‐week horizon. This approximation is adequate for comparing long‐term mechanistic scenarios but does not capture intra‐dosing‐interval PK peaks and troughs. This limitation is most relevant for rapamycin, where intermittent regimens may generate on‐mTOR/off‐mTOR cycling with a safety profile distinct from chronic continuous exposure. Approximates monthly pulsed repair induction and shows attenuated but directionally preserved improvement in BAG.

### 4.5 Clinical Safety Context for Combination Therapies

The rapamycin‐containing combination is a theoretical mechanistic construct, not a clinical recommendation. Chronic mTOR inhibition may carry risks of immunosuppression, dyslipidaemia, impaired wound healing, infection susceptibility and gastrointestinal intolerance. Rapamycin is also metabolized by CYP3A4 and is a P‐gp substrate; GLP‐1RA‐associated delayed gastric emptying could plausibly affect oral rapamycin absorption. These safety issues require clinical evaluation before any therapeutic translation.

### 4.6 Regulatory Landscape and Model Credibility

Aging is not currently an FDA‐ or EMA‐approved therapeutic indication. The appropriate regulatory framing is therefore not submission for an aging indication, but mechanism‐based prioritization for trials targeting accepted age‐related outcomes such as frailty, multimorbidity, physical function or conventional cardiometabolic endpoints with exploratory aging biomarkers. FDA model‐informed drug development programmes, ICH M15 and ASME V&V40 provide useful documentation and credibility frameworks, but they do not make the present model regulatory‐ready [40, 41, 42].

### 4.7 Model Limitations, Quantitative Sensitivity Bounds, and External Validation Requirements

The most important limitation is the absence of a reserved hold‐out clinical dataset. The STEP endpoints and literature‐derived anchors were used for model calibration and subsequent internal consistency checks; they do not constitute independent validation. No external clinical validation was performed. The aging layer also relies on surrogate population anchors rather than individual‐level longitudinal trajectories, so the model should be considered a calibrated hypothesis‐generation tool rather than a validated predictor.

For sarcopenia, the model predicts 52‐week body‐weight changes of −16% for the metabolic combination and −13% for the aging‐oriented combination. Applying f_LM = 0–0.40 gives an unmodelled lean‐mass‐loss bound of 0%–6.4% and 0%–5.2% of baseline body mass, respectively. Because the present frailty equation contains no lean‐mass or muscle‐function penalty, the simulated frailty improvements (−8% and −13%) should be interpreted as upper‐bound benefits. Attenuation, loss of benefit, or rank reversal in older or sarcopenic populations cannot be excluded.

For epigenetic aging, BAG is a dimensionless damage‐repair state and has not been calibrated to a DNA‐methylation clock. Under the transfer‐slope bound s_epi = 0.5–1.5, the metabolic‐arm BAG change of −11% maps to −5.5% to −16.5% on a normalized transferred scale, whereas the aging‐oriented change of −20% maps to −10% to −30%; these intervals overlap. The ±3.6‐year clock error is applied only after an empirical mapping from normalized BAG to years is estimated and must not be numerically combined with the present percentage outputs. Consequently, the mechanistic rank ordering may be used for hypothesis generation, but absolute epigenetic‐age change and between‐arm separation remain unvalidated (Table 3).

**TABLE 3:** Quantitative sensitivity bounds for omitted sarcopenia and epigenetic‐age domains.

Omitted domain	Sensitivity bound	Model‐implied range	Interpretation
Lean mass/sarcopenia	f_LM = 0–0.40 of total weight loss [38]	Metabolic arm: 0%–6.4% baseline mass; aging arm: 0%–5.2%	FRA improvements are upper bounds; sarcopenic rank reversal cannot be excluded.
Epigenetic age gap	s_epi = 0.5–1.5; post‐mapping error ±3.6 years [39]	Normalized BAG: −5.5% to −16.5% versus. −10% to −30%	Absolute age‐equivalent change and arm separation are not validated.

External validation against independent semaglutide, SGLT2 inhibitor combination, rapamycin/rapalogue, body‐composition, functional‐frailty, and aging‐biomarker datasets is therefore required. A virtual‐patient population is also needed to represent sex, baseline age, frailty, muscle mass, renal function, metabolic reserve, and epigenetic‐clock variability.

### 4.8 Future Directions

Future model versions should incorporate explicit rapamycin PK, sodium‐fluid and renal hemodynamic SGLT2i mechanisms, lean‐mass‐to‐frailty coupling, virtual‐patient variability, individual‐level longitudinal biomarker data and prospective validation against independent datasets. These extensions would move the model from mechanistic prioritization toward trial design and sample‐size estimation for healthspan‐directed pharmacology.

## 5 Conclusion

The revised model supports a transition from disease‐centered pharmacology toward hypothesis‐generating, aging‐aware therapeutic design. GLP‐1 RA + SGLT2 inhibitor + metformin emerges as the stronger metabolic combination, whereas GLP‐1 RA + SGLT2 inhibitor + rapamycin emerges as the stronger repair‐ and aging‐oriented mechanistic hypothesis. These optima are distinct because metabolic improvement and aging modulation are only partially overlapping systems objectives. The findings should be interpreted as model‐based hypotheses requiring external validation, population‐variability modeling and safety evaluation rather than as treatment recommendations.

## Author Contributions

Igor Goryanin and Irina Goryanin wrote the manuscript; Igor Goryanin designed the research; Igor Goryanin performed the research; Igor Goryanin and Bob Damms analyzed the data.

## Funding

The authors have nothing to report.

## Ethics Statement

The authors have nothing to report.

## Conflicts of Interest

All authors are affiliated with IQANOVA Ltd., which develops AI‐enabled QSP platforms.

## Supporting information


**Figure S1:** Parameter identifiability: Cramér–Rao RSE and FIM correlation matrix.
**Figure S2:** Biomarker trajectories under single‐drug treatment.
**Figure S3:** Comprehensive model fit overview across calibration targets.
**Table S1:** Model calibration performance across all endpoints.
**Table S2:** Global sensitivity analysis: Spearman rank correlation.

## Data Availability

The SBML model (IQANOVA_GLP1_Ageing_QSP.xml), synthetic calibration dataset (all_synthetic_data.csv), GSA results, Bayesian meta‐analysis code, identifiability reports and supplementary figures/tables are included with the Supporting Information. On acceptance, the SBML/COMBINE archive will be deposited in BioModels and mirrored at the IQANOVA ATLAS (www.iqanova.org); persistent accession links will be added at proof stage.
