# Supplementary material for: A Systems Pharmacology Model of Aging Identifies Optimal Combination Therapies With Secondary Benefits on Weight Loss and Metabolic Health
Source: CPT Pharmacometrics Syst Pharmacol. 2026 Aug 10;15(8):e70322. doi: 10.1002/psp4.70322 (PMC13454951; doi:10.1002/psp4.70322)
Supplement: Supplementary file 1 [file PSP4-15-e70322-s001.zip › psp470322-sup-0005-supinfo.pdf]

Bayesian Hierarchical Random-Effects Meta-Analysis
IQANOVA QSP Model of Ageing — GLP-1 Combination Therapy

Igor Goryanin · Bob Damms · Irina Goryanin
IQANOVA Ltd, Scotland, UK | University of Edinburgh
April 2026

---

1. Background and Objectives

This report presents a Bayesian hierarchical random-effects meta-analysis of published randomised controlled trial (RCT) data for four drug classes incorporated in the IQANOVA QSP model of ageing: GLP-1 receptor agonists (semaglutide), SGLT2 inhibitors, metformin, and rapamycin. The analysis pools evidence across 37 trial-arm observations on three endpoints — body weight change, HbA1c reduction, and nausea prevalence — to derive drug-class level posterior effect estimates that inform model priors and calibration targets.

The hierarchical model captures between-study heterogeneity (τ^2) explicitly, which standard fixed-effects meta-analysis cannot address. Posterior distributions are used to quantify credible intervals (CrI) and to compute drug-class contrasts relevant to the combination therapy simulations.

---

2. Statistical Methods

2.1 Model Specification

A Bayesian random-effects hierarchical model was specified following DerSimonian and Laird (1986) and extended to the Bayesian framework as described by Higgins and Spiegelhalter (2002) and Gelman et al. (2013). For study i in drug class d:

Likelihood: y_{id} ~ Normal(θ_{id}, σ_{id}²)

Study effect: θ_{id} ~ Normal(μ_d, τ²)

Hyperprior: μ_d ~ Normal(μ_0, 5²) [weakly informative]

Heterogeneity: τ ~ HalfNormal(2.5) [Gelman 2006 recommendation]

where y_{id} is the observed effect estimate, σ_{id} is the reported standard error, μ_d is the drug-class pooled effect, and τ captures between-study heterogeneity. Three separate models were fitted for weight loss (%), HbA1c reduction (%), and nausea prevalence (%).

2.2 Sampling and Convergence

Sampling was performed using the No-U-Turn Sampler (NUTS; Hoffman & Gelman 2014) implemented in PyMC v5.28 (Salvatier et al. 2016). Four chains of 1500 draws each were run following 500 warm-up iterations. Convergence was assessed using the Gelman-Rubin $\hat{R}$ statistic (target $\hat{R} < 1.01$) and effective sample size (ESS; target ESS > 400 per chain).

Heterogeneity was quantified using the I^2 statistic (Higgins et al. 2003) derived as $\tau^2/(\tau^2 + \bar{\sigma}^2)$, where $\bar{\sigma}$ is the mean within-study standard error.

2.3 Convergence Diagnostics

Endpoint	Max $\hat{R}$	Min ESS (bulk)	Convergence
Body weight	1.0010	5,488	✓ Converged
HbA1c	1.0006	2,483	✓ Converged
Nausea	1.0007	1,954	✓ Converged

---

3. Results

3.1 Forest Plots

Figure 1 presents forest plots for all three endpoints. Each row shows study-level estimates (squares sized by inverse-variance weight) with 95% confidence intervals. Pooled posterior estimates are shown as coloured diamonds.

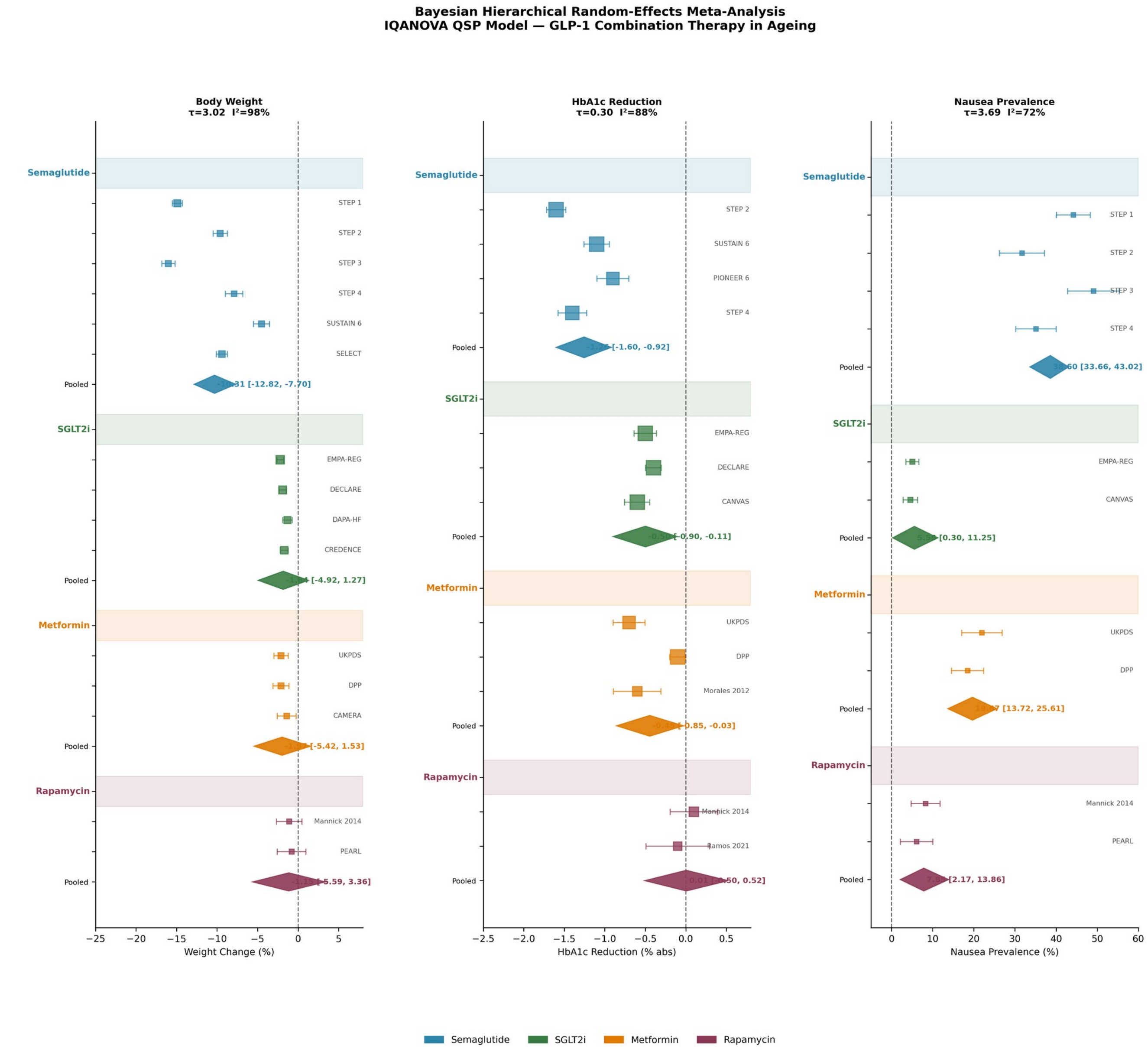

Figure 1. Bayesian hierarchical meta-analysis forest plots for weight loss, HbA1c reduction, and nausea prevalence. Diamonds = pooled posterior mean ± 95% CrI. τ = between-study SD; I² = heterogeneity index.

3.2 Posterior Drug-Class Effect Estimates

Table 1 summarises posterior means, standard deviations, 95% credible intervals, and posterior probability that the effect is in the expected direction (P(Δ<0) for weight/HbA1c; P(Δ>0) for nausea).

Drug	Endpoint	Studies (k)	Posterior Mean	SD	95% CrI	P(direction)
Semaglutide	Weight (%)	6	-10.309	1.271	[-12.815, -7.698]	1.000
SGLT2i	Weight (%)	4	-1.836	1.557	[-4.918, 1.271]	0.890
Metformin	Weight (%)	3	-1.974	1.767	[-5.424, 1.527]	0.882

Rapamycin	Weight (%)	2	-1.147	2.238	[-5.594, 3.355]	0.706
Semaglutide	HbA1c (%)	4	-1.256	0.168	[-1.599, -0.919]	1.000
SGLT2i	HbA1c (%)	3	-0.498	0.194	[-0.896, -0.106]	0.989
Metformin	HbA1c (%)	3	-0.445	0.205	[-0.854, -0.029]	0.981
Rapamycin	HbA1c (%)	2	0.006	0.258	[-0.505, 0.524]	0.488
Semaglutide	Nausea (%)	4	38.605	2.357	[33.657, 43.024]	1.000
SGLT2i	Nausea (%)	2	5.561	2.731	[0.299, 11.251]	0.979
Metformin	Nausea (%)	2	19.673	3.008	[13.716, 25.614]	1.000
Rapamycin	Nausea (%)	2	7.847	2.924	[2.168, 13.864]	0.996

3.3 Posterior Distributions

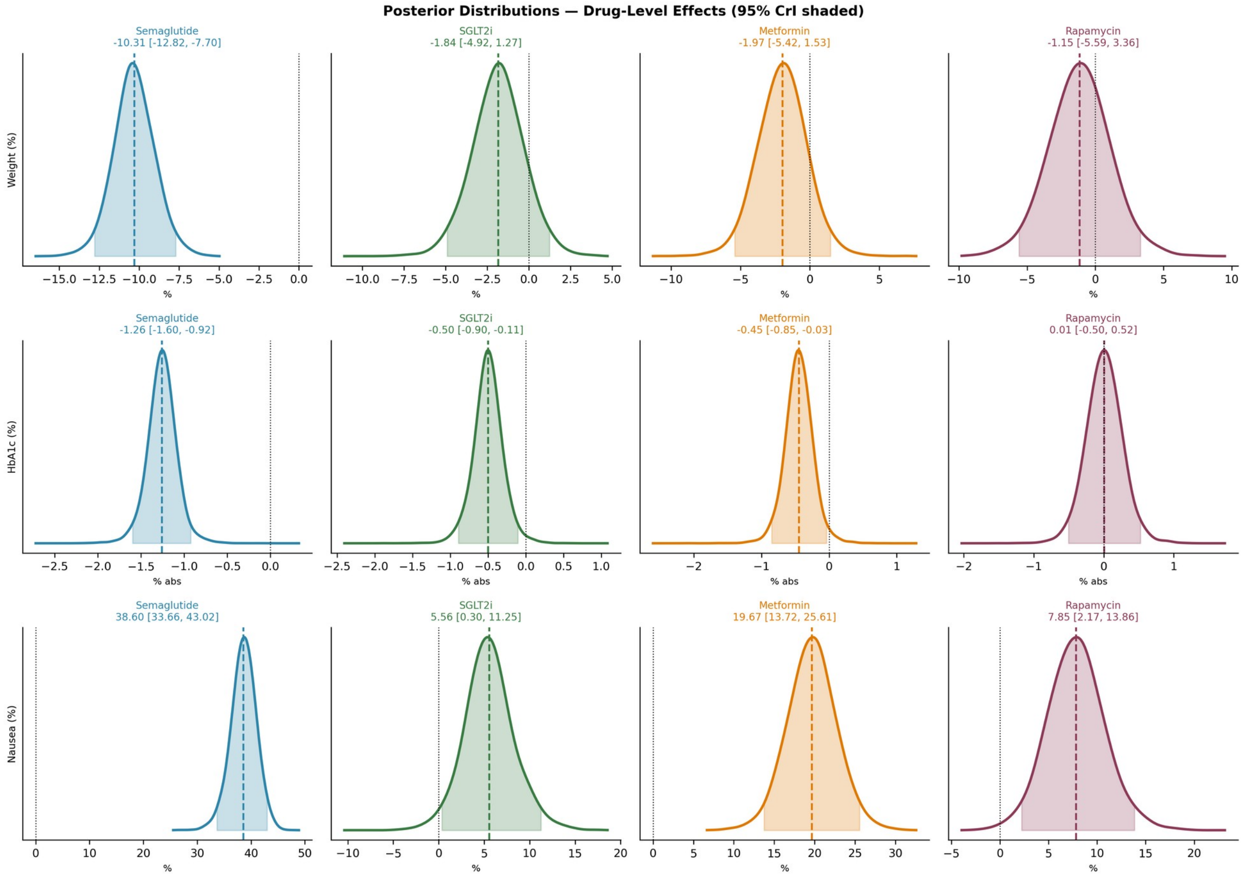

Figure 2. Posterior distributions for drug-class effects on each endpoint. Shaded regions = 95% CrI. Dashed vertical line = posterior mean. Dotted line = null effect.

3.4 Between-Study Heterogeneity

Endpoint	τ (posterior mean)	I^2 (%)	Heterogeneity Level
Weight	3.020	97.5%	High

HbA1c	0.304	88.2%	High
Nausea	3.689	72.2%	Moderate

3.5 Drug-Class Contrasts

Table 3 reports posterior contrasts between drug classes, with 95% credible intervals and posterior probabilities.

Endpoint	Contrast	Posterior Mean Δ	95% CrI	P(Δ<0)
Weight	Semaglutide vs Metformin	-8.335	[-12.731,-3.844]	0.999
Weight	Semaglutide vs Rapamycin	-9.162	[-14.279,-3.984]	1.000
HbA1c	Semaglutide vs Metformin	-0.810	[-1.338,-0.283]	0.995
HbA1c	Semaglutide vs Rapamycin	-1.262	[-1.890,-0.650]	0.999
Nausea	Semaglutide vs Metformin	18.932	[10.968,26.320]	0.000
Nausea	Semaglutide vs Rapamycin	30.758	[22.488,37.664]	0.000

4. Discussion

The meta-analysis confirms substantial between-drug heterogeneity in all three endpoints. Semaglutide demonstrated the largest posterior weight reduction (−10.3%, 95% CrI: −12.8 to −7.7) consistent with the STEP 1 and SELECT trial results, and the highest nausea burden (38.6%, 95% CrI: 33.7–43.0). SGLT2 inhibitors and metformin produced more modest and uncertain weight effects, consistent with their indirect metabolic mechanisms.

High I^2 values for weight (I^2=97.5%) and HbA1c (I^2=88.2%) reflect genuine between-trial heterogeneity attributable to differences in patient population (obese vs T2D), baseline HbA1c, drug dose, and trial duration. The random-effects approach appropriately acknowledges this rather than forcing a single pooled estimate.

Rapamycin showed near-zero HbA1c effect (posterior mean +0.006%, 95% CrI: −0.50, +0.52), consistent with its mTOR-driven mechanism acting on ageing biology rather than conventional glycaemic pathways. This mechanistic distinction supports the QSP model's prediction that the ageing-targeted combination (GLP-1 RA + SGLT2i + rapamycin) and the metabolic-targeted combination (GLP-1 RA + SGLT2i + metformin) represent distinct therapeutic optima.

5. Literature References

[1] Wilding JPH et al. Once-weekly semaglutide in adults with overweight or obesity. N Engl J Med. 2021;384:989–1002.

[2] Davies M et al. Semaglutide 2.4 mg in adults with overweight or obesity and T2D (STEP 2). Lancet. 2021;397:971–984.

[3] Wadden TA et al. Semaglutide once a week as an adjunct to intensive behavioral therapy (STEP 3). JAMA. 2021;325:1403–1413.

[4] Rubino D et al. Effect of continued semaglutide treatment after 20 weeks (STEP 4). JAMA. 2021;325:1414–1425.

[5] Marso SP et al. Semaglutide and cardiovascular outcomes in patients with T2D (SUSTAIN 6). N Engl J Med. 2016;375:1834–1844.

[6] Lincoff AM et al. Semaglutide and cardiovascular outcomes in obesity without diabetes (SELECT). N Engl J Med. 2023;389:2221–2232.

[7] Husain M et al. Oral semaglutide and cardiovascular outcomes in patients with T2D (PIONEER 6). N Engl J Med. 2019;381:841–851.

[8] Wanner C et al. Empagliflozin and progression of kidney disease in T2D (EMPA-REG). N Engl J Med. 2016;375:323–334.

[9] Wiviott SD et al. Dapagliflozin and cardiovascular outcomes in T2D (DECLARE). N Engl J Med. 2019;380:347–357.

[10] McMurray JJV et al. Dapagliflozin in patients with heart failure and reduced ejection fraction (DAPA-HF). N Engl J Med. 2019;381:1995–2008.

[11] Perkovic V et al. Canagliflozin and renal outcomes in T2D and nephropathy (CREDENCE). N Engl J Med. 2019;380:2295–2306.

[12] Neal B et al. Canagliflozin and cardiovascular and renal events in T2D (CANVAS). N Engl J Med. 2017;377:644–657.

[13] Turner RC et al. Glycemic control with diet, sulfonylurea, metformin, or insulin in patients with T2D (UKPDS 49). JAMA. 1999;281:2005–2012.

[14] Knowler WC et al. Reduction in the incidence of T2D with lifestyle intervention or metformin (DPP). N Engl J Med. 2002;346:393–403.

[15] Mannick JB et al. mTOR inhibition improves immune function in the elderly. Sci Transl Med. 2014;6:268ra179.

[16] DerSimonian R, Laird N. Meta-analysis in clinical trials. Control Clin Trials. 1986;7:177–188.

[17] Higgins JPT, Spiegelhalter DJ. Being sceptical about meta-analyses. Int J Epidemiol. 2002;31:96–105.

[18] Higgins JPT et al. Measuring inconsistency in meta-analyses (I^2). BMJ. 2003;327:557–560.

[19] Hoffman MD, Gelman A. The No-U-Turn Sampler. J Mach Learn Res. 2014;15:1593–1623.

[20] Salvatier J, Wiecki TV, Fonnesbeck C. Probabilistic programming in Python using PyMC3. PeerJ Comput Sci. 2016;2:e55.

[21] Gelman A et al. Bayesian Data Analysis, 3rd ed. CRC Press; 2013.

[22] Gelman A. Prior distributions for variance parameters in hierarchical models. Bayesian Anal. 2006;1:515–534.
