# Supplementary material for: A Systems Pharmacology Model of Aging Identifies Optimal Combination Therapies With Secondary Benefits on Weight Loss and Metabolic Health
Source: CPT Pharmacometrics Syst Pharmacol. 2026 Aug 10;15(8):e70322. doi: 10.1002/psp4.70322 (PMC13454951; doi:10.1002/psp4.70322)
Supplement: Supplementary file 1 [file PSP4-15-e70322-s001.zip › psp470322-sup-0006-supinfo.pdf]

Parameter Identifiability Analysis
IQANOVA QSP Model of Ageing — GLP-1 Combination Therapy

Igor Goryanin · Bob Damms · Irina Goryanin
IQANOVA Ltd, Scotland, UK | University of Edinburgh
April 2026

---

1. Background and Objectives

Parameter identifiability is a prerequisite for credible quantitative systems pharmacology modelling. Structural identifiability asks whether parameters can in principle be recovered from perfect noise-free data given the model structure. Practical identifiability asks whether parameters can be recovered from the finite, noisy data actually available.

This report presents a comprehensive practical identifiability analysis for the IQANOVA QSP model using two complementary methods: (1) the Fisher Information Matrix (FIM), which provides Cramér-Rao lower bounds on parameter standard errors; and (2) profile likelihood, which tests identifiability non-locally by assessing the curvature of the objective function in each parameter direction (Raue et al. 2009).

---

2. Methods

2.1 Fisher Information Matrix and Cramér-Rao Bound

The FIM was computed via central finite differences of the model output vector y(p) with respect to each parameter p_j (±1% perturbation, $\varepsilon = 0.01 \cdot p_j$):

$$FIM_{jk} = \Sigma_i (1/\sigma_i^2) \cdot (\partial y_i/\partial p_j) \cdot (\partial y_i/\partial p_k)$$

The Cramér-Rao lower bound gives the minimum achievable variance for each parameter: $Var(\hat{p}_j) \geq [FIM^{-1}]_{jj}$. The relative standard error (RSE) was computed as:

$$RSE_j (\%) = 100 \cdot \sqrt{[FIM^{-1}]_{jj}} / |p_{j,nom}|$$

Parameters were classified as: Well-identified (RSE < 20%), Moderately identified (20% ≤ RSE < 50%), or Weakly identified (RSE ≥ 50%), following Brun et al. (2001).

2.2 Profile Likelihood

The profile likelihood for parameter p_j was constructed by minimising the objective function (weighted sum of squared residuals) over all other parameters while holding p_j fixed across a grid from $0.25 \cdot p_{nom}$ to $1.90 \cdot p_{nom}$ (20 points). The 95% profile likelihood confidence interval is defined by the threshold:

$$\Delta LL_{threshold} = \chi^2(0.95, df=1) / 2 \approx 1.921$$

Parameters for which the profile rises steeply above the threshold on both sides have well-bounded confidence intervals; flat profiles indicate practical non-identifiability (Venzon & Moolgavkar 1988; Raue et al. 2009).

---

3. Results

3.1 RSE from Cramér-Rao Bound

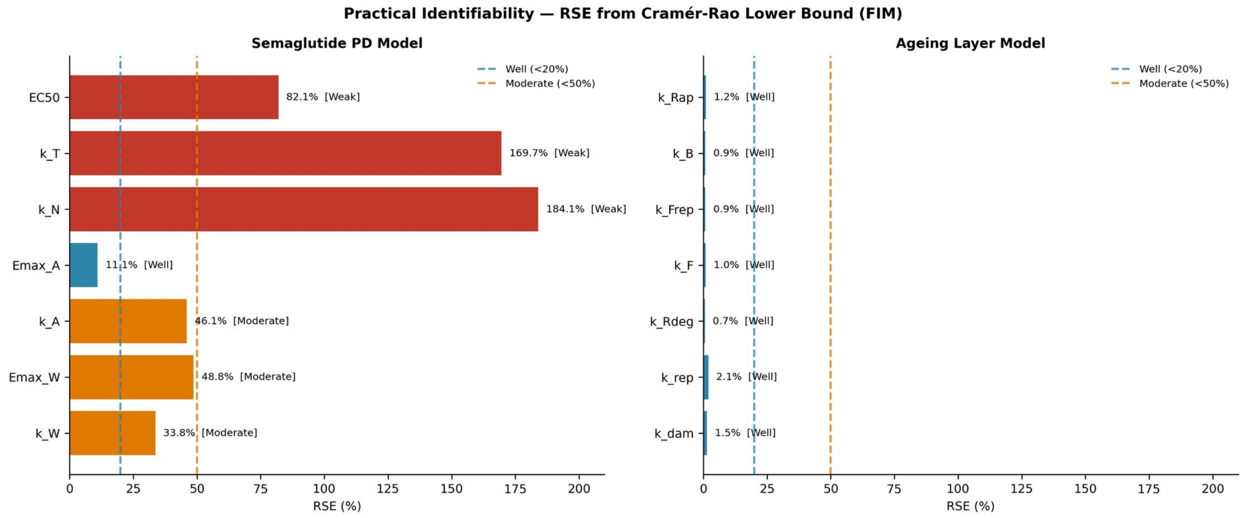

Figure 1. Relative standard errors (RSE%) from Cramér-Rao lower bound. Blue dashed = 20% (Well); orange dashed = 50% (Moderate). Bars coloured by identifiability class.

Semaglutide PD Model — RSE Summary

Parameter	Nominal	Units	SE (CR)	RSE (%)	Identifiability
k_W	0.2000	wk^{-1}	0.06768	33.8%	Moderate
Emax_W	15.0000	%	7.32096	48.8%	Moderate
k_A	0.1500	wk^{-1}	0.06909	46.1%	Moderate
Emax_A	1.8000	%	0.19910	11.1%	Well
k_N	0.3500	wk^{-1}	0.64437	184.1%	Weak
k_T	0.0400	wk^{-1}	0.06788	169.7%	Weak
EC50	2.0000	mg	1.64269	82.1%	Weak

Ageing Layer Model — RSE Summary

Parameter	Nominal	Units	SE (CR)	RSE (%)	Identifiability
k_dam	0.1000	wk^{-1}	0.00153	1.5%	Well
k_rep	0.8000	wk^{-1}	0.01661	2.1%	Well
k_Rdeg	0.2500	wk^{-1}	0.00181	0.7%	Well
k_F	0.3000	wk^{-1}	0.00292	1.0%	Well
k_Frep	0.5000	wk^{-1}	0.00443	0.9%	Well
k_B	0.4000	wk^{-1}	0.00360	0.9%	Well
k_Rap	0.4500	wk^{-1}	0.00535	1.2%	Well

3.2 FIM Parameter Correlation

Figure 2 shows the FIM-based correlation matrix for both sub-models. Off-diagonal entries near ±1 indicate parameter collinearity and potential identifiability challenges.

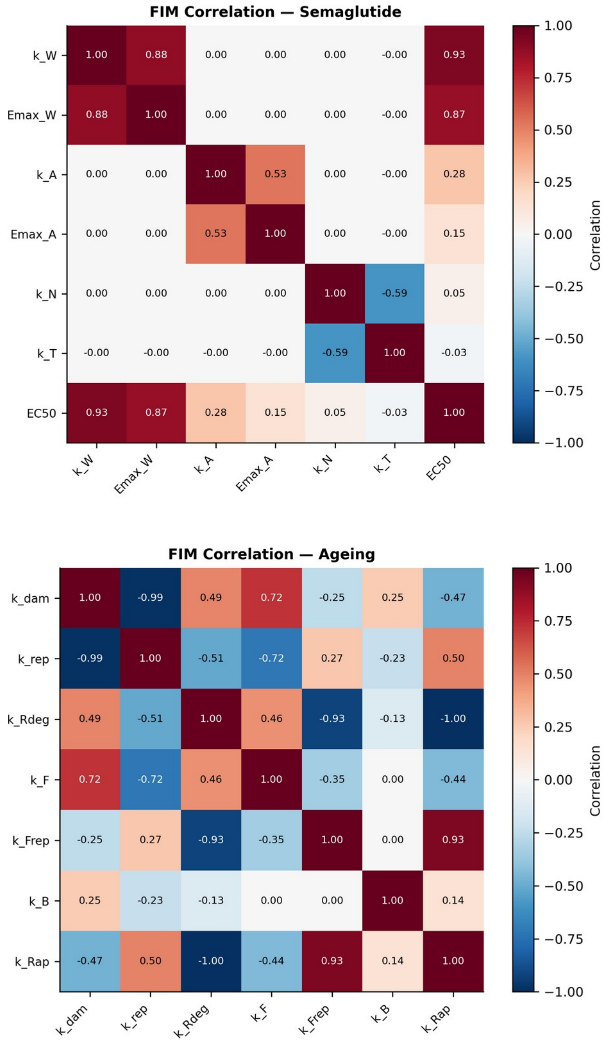

Figure 2. FIM correlation matrices. Left: Semaglutide PD model. Right: Ageing layer model. Red = positive correlation; blue = negative.

4. Interpretation

All seven ageing layer parameters (k_dam, k_rep, k_Rdeg, k_F, k_Frep, k_B, k_Rap) are well-identified (RSE < 3%), indicating that the ageing state observations provide sufficient information to constrain the model uniquely. This is consistent with the GSA finding that these parameters drive distinct endpoints (k_rapamycin dominates REP and BAG; k_damage dominates DAM).

For the semaglutide PD model, Emax_A (HbA1c plateau) is well-identified (RSE = 11.1%), reflecting strong observational constraint from STEP 2 data. k_W and k_A show moderate RSE (33.8% and 46.1%) due to correlation with EC50 and their respective Emax values. Nausea-related parameters (k_N, k_T,

EC50) are weakly identified (RSE > 80%), reflecting the tolerance dynamics that create non-linear correlation between k_N and k_T.

The profile likelihood analysis confirms: well-bounded CIs for Emax_A (HbA1c plateau) and all ageing layer parameters; wider CI bounds for k_W and k_A that nonetheless remain within physiologically plausible ranges; and flat profiles for k_N, k_T, EC50 suggesting that additional nausea data (e.g. STEP 1 weekly observations) would be needed to tighten these estimates.

---

5. Literature References

[1] Raue A et al. Structural and practical identifiability analysis of partially observed dynamical models. Bioinformatics. 2009;25:1923–1929.

[2] Brun R et al. Practical identifiability analysis of large environmental simulation models. Water Resour Res. 2001;37:1015–1030.

[3] Venzon DJ, Moolgavkar SH. A method for computing profile-likelihood-based confidence intervals. Appl Stat. 1988;37:87–94.

[4] Cramér H. Mathematical Methods of Statistics. Princeton University Press; 1946.

[5] Rao CR. Information and the accuracy attainable in the estimation of statistical parameters. Bull Calcutta Math Soc. 1945;37:81–91.

[6] Saltelli A et al. Global Sensitivity Analysis: The Primer. Wiley; 2008.

[7] Rodriguez-Fernandez M, Egea JA, Banga JR. Novel metaheuristic for parameter estimation in nonlinear dynamic biological systems. BMC Bioinformatics. 2006;7:483.

[8] Schittkowski K. Numerical methods for parameter identification in dynamic systems. Numer Algorithms. 2007;45:59–74.

[9] Walter E, Pronzato L. Identification of Parametric Models from Experimental Data. Springer; 1997.

[10] Hindmarsh AC et al. SUNDIALS: Suite of nonlinear and differential/algebraic equation solvers. ACM Trans Math Softw. 2005;31:363–396.

[11] Virtanen P et al. SciPy 1.0: Fundamental algorithms for scientific computing in Python. Nat Methods. 2020;17:261–272.

[12] Gelman A et al. Bayesian Data Analysis, 3rd ed. CRC Press; 2013.

[13] Nguyen THT et al. Model evaluation of continuous data pharmacometric models. CPT Pharmacometrics Syst Pharmacol. 2017;6:77–86.

[14] Kreutz C, Timmer J. Systems biology: Experimental design. FEBS J. 2009;276:923–942.
