# Supplementary material for: A Systems Pharmacology Model of Aging Identifies Optimal Combination Therapies With Secondary Benefits on Weight Loss and Metabolic Health
Source: CPT Pharmacometrics Syst Pharmacol. 2026 Aug 10;15(8):e70322. doi: 10.1002/psp4.70322 (PMC13454951; doi:10.1002/psp4.70322)
Supplement: Supplementary file 1 [file PSP4-15-e70322-s001.zip › psp470322-sup-0001-supinfo.docx]

**Supplementary Materials**

**A Systems Pharmacology Model of Ageing Identifies Optimal Combination Therapies With Secondary Benefits on Weight Loss and Metabolic Health**

Igor Goryanin, Bob Damms, Irina Goryanin

Prepared for CPT: Pharmacometrics & Systems Pharmacology | Manuscript PSP-2026-0140

**Supplementary contents**

Figure S1. Parameter identifiability: Cramér–Rao RSE and FIM correlation matrix.

Figure S2. Biomarker trajectories under single-drug treatment.

Figure S3. Comprehensive model fit overview across calibration targets.

Table S1. Model calibration performance across all endpoints.

Table S2. Global sensitivity analysis: Spearman rank correlation.

**Figure S1**

Supplementary Figure S1. Parameter identifiability: Cramér–Rao RSE and FIM correlation matrix. (A) Relative standard error (RSE, %) for semaglutide pharmacodynamic parameters. (B) RSE for ageing-layer parameters. (C) Fisher Information Matrix correlation matrix for the ageing-layer parameter set. RSE, relative standard error; FIM, Fisher Information Matrix; PD, pharmacodynamics.

**Figure S2**

Supplementary Figure S2. Biomarker trajectories under single-drug treatment over 52 weeks. Synthetic data are shown as circles with standard-error bars, and model fit curves are shown as dashed lines with standard-error bands. Drug classes: semaglutide, metformin, SGLT2 inhibitor, and rapamycin. GDF15, growth differentiation factor 15; eGDR, estimated glucose disposal rate; SGLT2i, sodium-glucose cotransporter-2 inhibitor; SE, standard error.

**Figure S3**

Supplementary Figure S3. Comprehensive model fit overview across all calibration targets. Clinical response, biomarker trajectories, and ageing-state simulations are shown across the full model. DAM, damage accumulation; BAG, biological age gap; GDF15, growth differentiation factor 15.

**Table S1**

Table S1. Model calibration performance across all endpoints.

| **Endpoint** | **Arm** | **RMSE** | **Unit** | **Benchmark** | **Source** |
| --- | --- | --- | --- | --- | --- |
| Body weight change | 1 mg | 0.637 | % | < 1.0% | STEP 2, Davies 2021 |
| Body weight change | 2.4 mg | 0.543 | % | < 1.0% | STEP 1, Wilding 2021 |
| HbA1c reduction | 1 mg | 0.039 | % abs | < 0.15% | STEP 2, Davies 2021 |
| HbA1c reduction | 2.4 mg | 0.036 | % abs | < 0.15% | STEP 2, Davies 2021 |
| Nausea prevalence | 1 mg | 1.741 | % patients | < 5% | STEP 2, Davies 2021 |
| Nausea prevalence | 2.4 mg | 1.360 | % patients | < 5% | STEP 1, Wilding 2021 |
| DAM (baseline combo) | — | 1.341 | a.u. ×100 | < 3.0 | Literature synthesis |
| DAM (ageing combo) | — | 0.713 | a.u. ×100 | < 3.0 | Literature synthesis |
| Repair (ageing combo) | — | 1.210 | a.u. ×100 | < 3.0 | Literature synthesis |
| BioAgeGap (ageing combo) | — | 1.299 | a.u. ×100 | < 3.0 | Literature synthesis |

**Table S2**

Table S2. Global sensitivity analysis: Spearman rank correlation. N = 3,000 Latin Hypercube Sampling realizations, ±30% parameter variation. *** p < 0.001; — = |rho| < 0.10.

| **Parameter** | **Weight 68 wk** | **HbA1c 68 wk** | **Nausea peak** | **DAM 52 wk** | **Repair 52 wk** | **BAG 52 wk** | **Parameter space** |
| --- | --- | --- | --- | --- | --- | --- | --- |
| k_weight | −0.879*** | — | — | — | — | — | Metabolic |
| k_HbA1c | — | −0.877*** | — | — | — | — | Metabolic |
| k_nausea | — | — | +0.658*** | — | — | — | Tolerability |
| k_tolerance | — | — | −0.643*** | — | — | — | Tolerability |
| EC50 | −0.466*** | −0.469*** | +0.365*** | — | — | — | Shared |
| k_damage | — | — | — | +0.520*** | — | +0.133*** | Ageing |
| k_repair_clear | — | — | — | −0.487*** | — | −0.094*** | Ageing |
| k_repair_decay | — | — | — | +0.491*** | −0.689*** | +0.593*** | Ageing |
| k_rapamycin | — | — | — | −0.469*** | +0.689*** | −0.631*** | Ageing (key) |
| k_BioAgeGap | — | — | — | — | — | −0.416*** | Ageing |

*Note: Supplementary numbering corresponds to items moved from the original manuscript file to comply with the CPT:PSP limit of seven total figures and tables in the main article.*
